# Cognitive vulnerability and dental fear

**DOI:** 10.1186/1472-6831-8-2

**Published:** 2008-01-24

**Authors:** Jason M Armfield, Gary D Slade, A John Spencer

**Affiliations:** 1 Australian Research Centre for Population Oral Health, School of Dentistry, University of Adelaide, South Australia, Australia

## Abstract

**Background:**

The Cognitive Vulnerability Model proposes that perceptions of certain characteristics of a situation are critical determinants of fear. Although the model is applicable to all animal, natural environment and situational fears, it has not yet been applied specifically to dental fear. This study therefore aimed to examine the association between dental fear and perceptions of dental visits as uncontrollable, unpredictable and dangerous.

**Methods:**

The study used a clustered, stratified national sample of Australians aged 15 years and over. All participants were asked in a telephone interview survey to indicate their level of dental fear. Participants who received an oral examination were subsequently provided with a self-complete questionnaire in which they rated their perceptions of uncontrollability, unpredictability and dangerousness associated with dental visiting.

**Results:**

3937 participants were recruited. Each of the three vulnerability-related perceptions was strongly associated with the prevalence of high dental fear. In a logistic regression analysis, uncontrollability and dangerousness perceptions were significantly associated with high dental fear after controlling for age and sex. However, unpredictability perceptions did not have a statistically significant independent association with dental fear after controlling for all other variables.

**Conclusion:**

Results are mostly consistent with the Cognitive Vulnerability Model of the etiology of fear, with perceptions of uncontrollability, unpredictability and dangerousness each showing a strong bivariate relationship with high dental fear prevalence. However, more extensive measures of vulnerability perceptions would be valuable in future investigations.

## Background

People with high dental fear and dental phobias often experience a range of aversive psychological, emotional and social problems [1]. Although dental fear is a diagnosable psychological condition with associated psychological symptoms [2], it also has important and challenging physical health implications. People with dental fear often have poorer oral health than people with no dental fear [3-6], and in some cases the long-term deferment of dental treatment may lead to the development of oral pain and the need for invasive and potentially painful dental treatment. Indeed, research has consistently shown that people with dental fear are more likely to delay dental appointments [7,8] and there is some evidence that this may set up a vicious cycle of dental fear, whereby delayed dental visiting allows the continued progression of oral disease which may lead to the requirement for emergency treatment which then serves to exacerbate or maintain the person's dental fear [9-12]. Dental fear has a high prevalence in many western countries [13,14] yet it is the serious oral health consequences of dental fear that stand it apart from many other specific fears and which make it such an important area of study.

It is often assumed, by practitioners and the lay public alike, that dental fear is a result of having had a painful or unpleasant past experience associated with a dental examination or procedure. Consistent with this belief, many studies have found instances of aversive past dental experiences among dental phobics [15-19] and interviews with dental phobics often reveal numerous traumatic experiences, sometimes spanning decades [20]. Such a view is consistent with the classical conditioning model of the etiology of fear, which has its origins in studies of animals reacting to painful stimuli. While the conditioning model of the genesis of fear and its various subsequent revisions appear to account for some cases of dental fear, it does not account for a number of troublesome features of dental fear. First, many people have undergone dental treatment yet only some people develop dental fear [21]. Second, there are a number of people who have never had or can not recall a traumatic dental experience yet report being afraid of going to the dentist [22]. Finally, being afraid of the dentist appears to relate to a number of other fears [23-25], which we would not expect where it merely a function of learning experiences.

In an effort to address some of the more intractable issues related to explaining specific fears, Armfield has proposed a model of the etiology of fear which positions cognitions, rather than experiences, as the central element in fear acquisition and expression [26]. It is proposed that it is a person's perceptions of a stimulus or situation which are crucial in the etiology of fear. Specifically, perceptions of uncontrollability, unpredictability, dangerousness and disgustingness are argued to create a powerful feeling of vulnerability. Borrowing from cognitive psychological theory, vulnerability-related perceptions are believed to be incorporated into a schema, a cognitive structure that serves to filter information and guide experiences, beliefs, emotions and behaviours. An individual enters any given situation with a pre-existing schema, which serves to shape the behavioural, psychological and physiological experience of that situation.

**Figure 1:**
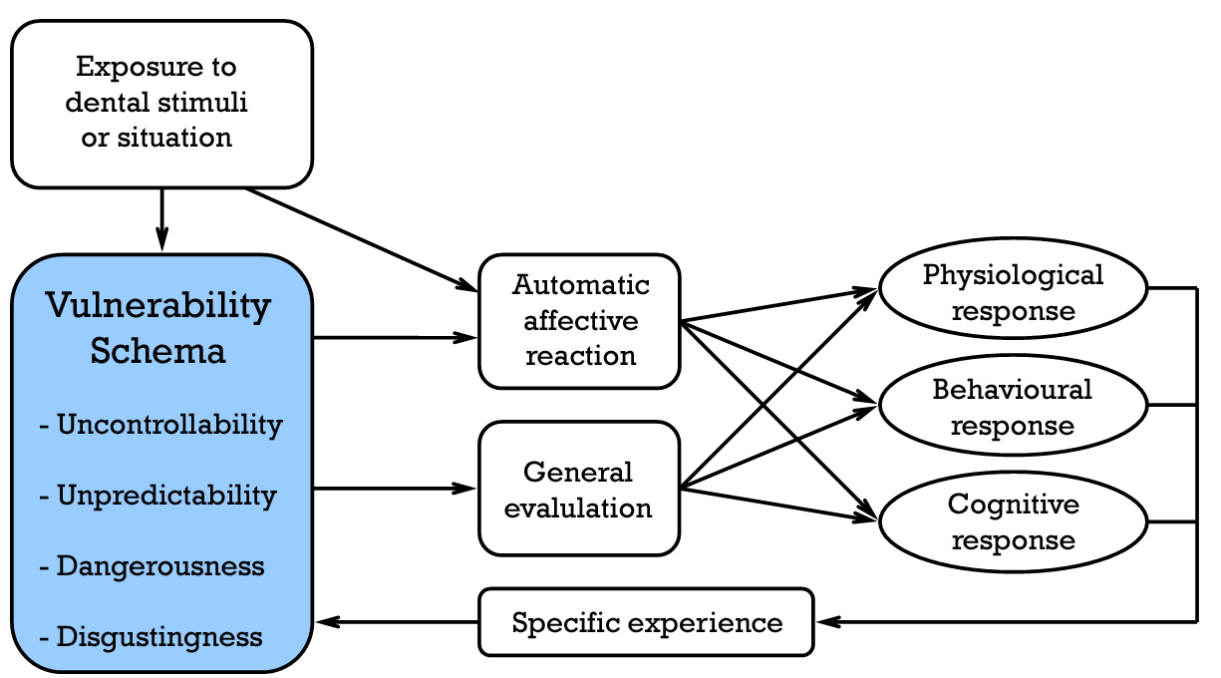
Cognitive Vulnerability Model of the elicitation of a fear response to dental stimuli.

A summary of the Cognitive Vulnerability Model as it relates to the elicitation of dental fear is provided in Figure 1. In line with the model, encountering a dental stimulus or situation invokes a rapid and pre-conscious automatic affective reaction which primes a susceptible individual for a flight or fight response. Simultaneously, a person's vulnerability schema is activated and this feeds into a slower and more cognitive general evaluation of the significance of the situation to the person. The general evaluation is also influenced by other cognitive factors such as coping mechanisms and attentional biases. Both the automatic affective reaction to the dental situation and the general evaluation give rise to a suite of physiological, behavioural and cognitive/emotional responses in a fearful person, which may include nervousness, panic, sweating, a strong desire to leave the situation, catastrophic thoughts, worry, panic etc. The dental visiting experience, as well as the associated perceptions and emotions, feed back into the vulnerability schema, affecting continued exposure to the fear-relevant stimulus and determining future reactions to visiting the dentist.

The Cognitive Vulnerability Model has received support from a number of studies investigating fear of animals. High correlations have been found between fear of a number of different animals and perceptions of those animals as uncontrollable, unpredictable, dangerous and disgusting [27,28]. Also, experimental manipulation of perceptions of spiders as uncontrollable, unpredictable and dangerous has been found to have a significant effect on fear of encountering a spider [29,30]. However, the Cognitive Vulnerability Model has not yet been investigated in relation to dental fear. The current study therefore aimed to provide a preliminary investigation of the association between fear of going to the dentist and perceptions of uncontrollability, unpredictability and dangerousness associated with dental visiting. It was hypothesised that vulnerability-related perceptions would be significantly associated with dental fear after controlling for other possible confounding variables.

## Methods

Study participants were from a larger computer assisted telephone interview (CATI) survey of the Australian public contacted as part of the National Survey of Adult Oral Health (NSAOH), conducted in Australia between 2004 and 2006 [31]. Those people who were offered and received an oral examination as part of the NSAOH were subsequently sent a self-complete questionnaire. Data on individuals who completed the questionnaire were matched to information gathered from the telephone interview component of the survey to obtain the data used in this study.

The sampling frame for the NSAOH comprised an electronic version of the Australian national telephone listings. Fifteen strata were created, with probability proportional to size selection. The strata entailed metropolitan and non-metropolitan areas of seven of the eight Australian states and territories (New South Wales, Victoria, Queensland, Western Australia, South Australia, Tasmania and the Northern Territory) plus the single stratum of the Australian Capital Territory. The primary sampling unit was postcode and the secondary sampling unit was household. Thirty households were selected per metropolitan postcode while 40 households were selected per non-metropolitan postcode. One adult (15+ years) per household was selected.

Prior to initiating the CATI, a primary approach letter was mailed to each selected household. Initial telephone contact identified whether or not the number represented a residential dwelling and allowed for the random selection of one adult aged 15+ years. At the completion of the CATI, people aged ≥ 15 years who reported having natural teeth were asked to attend a dental examination. However, 255 people residing in remote areas of Australia were excluded from the examination phase of the study because it was not logistical possible for examining teams to visit them.

Dental fear was assessed in the CATI by the global question "Would you feel afraid or distressed when going to the dentist?" with response categories being 'Not at all' (1), 'A little afraid or distressed' (2), 'Moderately afraid or distressed' (3), 'Very afraid or distressed' (4) and 'Extremely afraid or distressed' (5). The global dental fear question was based on the Dental Anxiety Question (DAQ) [32], but essentially represented a new measure. Three changes were made from the DAQ. First, the response categories were expanded to 5 to allow for a more fine-grained analysis of dental fear and more scope for differential responding at the high fear end of the scale. Second, the wording was changed from "afraid" for the DAQ to "afraid or distressed" to counter a possible reluctance for some people to indicate what might be considered to be a socially unacceptable emotion. Finally, the phrase "Are you afraid..." in the DAQ was altered to "Would you feel afraid..." in the current study, to orient the respondent towards a future dental visit.

Responses to the dental fear question were dichotomised to create categories labelled 'low fear' (little or no fear) and 'high fear' (moderate to extreme fear), for the purposes of multivariate analyses. Because there is no general agreement on what cut-points should be used to define high fear, and as a check on the robustness of results using the dichotomisation adopted here, analyses using a higher threshold for 'high fear' were also used, with these categories corresponding to no fear/a little/moderate afraid and very/extremely afraid.

The self-complete questionnaire was mailed to participants and contained one question each relating to perceptions of control, predictability and likelihood of harm or danger when at the dentist. The items were "I don't feel in control when I'm in the dental chair", "I don't feel like I know what's going to happen next when I'm in the dental chair" and "I believe I will be hurt when I'm in the dental chair" with possible responses ranging from 'Strongly disagree' (1) to 'Strongly agree' (5). Due to space restrictions in the questionnaire, an item concerning disgust was not included in the self-complete questionnaire.

Data were weighted by state/territory and metropolitan/non-metropolitan residence to correct for varying probability of selection. Post hoc adjustments to the weighting by age and sex also occurred. Final weights were computed so that the sample characteristics approximated those of the Australian population. To account for design effects associated with the complex sample design employed in the survey, data for this study were analysed using SPSS™ Version 13 Complex Samples.

The NSAOH was reviewed and approved by both the University of Adelaide Human Research Ethics Committee and the Australian Institute of Health and Welfare Ethics committee. The nature of the interview was explained to subjects at their time of selection and verbal consent was obtained prior to asking questions.

## Results

A total of 36,931 telephone numbers were randomly sampled from the 'electronic white pages' which contained 28,812 in-scope telephone numbers. Out-of-scope refers to disconnected numbers, business and fax/modem numbers. In all, 14,123 completed interviews were conducted, a response rate of 49%. Among interviewed subjects, 12,606 people satisfied the inclusion criteria for the dental examination. Completed dental examinations numbered 5,505 which represented 46.7% of participants considered in-scope for examination. From participants who had received an oral examination, questionnaire data were subsequently obtained on 3,937 Australians aged 15 years and over. This represents 31.2% of those people who completed the telephone interview and met the inclusion criteria.

The mean age of participants was 44.0 years (SD = 17.2) with an age range of 15–90 years old. Slightly more than half of the participants were female (52.2%). A comparison of the study sample characteristics with those of the Australian population as reported in the 2001 Census of Housing and Population are provided in Table 1. The only notable difference between the study sample and the Australia population was in terms of employment status, with a higher percentage of the study sample being unemployed than in the Australian population and a lower percentage being students or retired than in the Australian population.

**Table 1:** A comparison of the study (questionnaire) sample characteristics with those of the Australian population aged 15+ in 2001

	Study sample		Australia 2001^†^	
		
	n	%	n	%
Age				
15–24	593	15.1	2,566,346	17.3
25–39	1,104	28.0	4,154,821	28.0
40–64	1,703	43.3	5,764,729	38.8
65–79	456	11.6	1,784,824	12.0
80+	81	2.1	586,054	3.9
Sex				
Male	1,884	47.8	7,347,379	48.9
Female	2,053	52.2	7,690,960	51.1
Language spoken at home^a^				
English	3,521	89.4	15,013,965	84.0
Language other than English	416	10.6	2,853,851	16.0
Income^b^				
<$20,000	488	12.4	654,331	13.3
$20,000–$39,999	766	19.5	1,164,952	23.6
$40,000–$59,000	680	17.3	913,398	18.5
$60,000–$79,000	564	14.3	509,987	10.3
$80,000+	1,041	26.5	1,082,855	21.9
Missing	397	10.1	611,305	12.4
Employment status				
Unemployed	583	14.8	660,709	4.4
Part-time	870	22.1	2,689,709	18.1
Full-time	1,549	39.4	5,360,693	36.1
Student/retired	758	19.2	5,265,426	35.4
Missing	177	4.5	880,237	5.9

^† ^Based on the Australian Bureau of Statistics Census of Population and Housing for Australia, 2001.

^a ^Australian data uses entire population for Language Spoken at Home.

^b ^Income categories for Australian data are: <$20,748 (<$399/wk); $20,749–$41,548 ($400–$799/wk); $41,549–$62,348 ($800–$1,199/wk); $62,349–$77,948 ($1,200–$1,499/wk); >$77,949 (>$1,500/wk)

**Figure 2:**
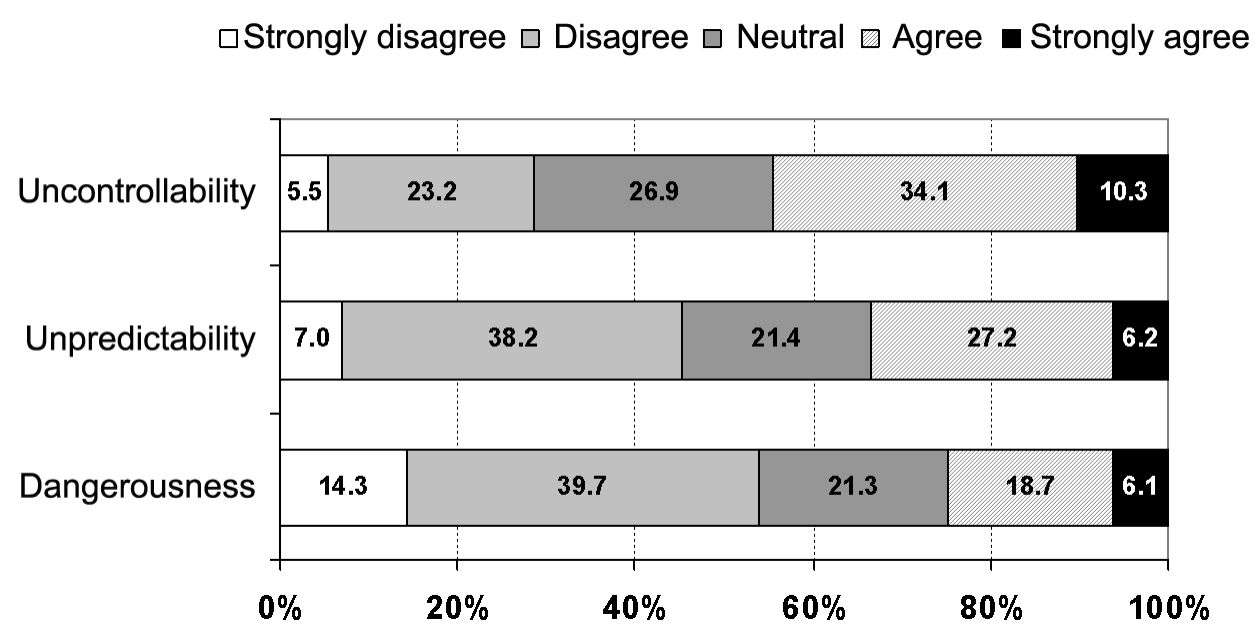
Distribution of responses regarding perceptions of uncontrollability, unpredictability and dangerousness associated with going to the dentist.

The majority of participants indicated that they felt no fear or distress when going to the dentist (57.5%). However, 21.9% of participants said that they were a little afraid or distressed, 11.8% that they were moderately afraid or distressed, 4.7% that they were very afraid or distressed, and 3.9% that they were extremely afraid or distressed. There were differences in the distribution of peoples' responses to how they felt when they were in the dental chair regarding perceptions of uncontrollability, unpredictability and dangerousness (Figure 2). Some 44.4% of people agreed or strongly agreed that they did not feel in control when in the dental chair, 33.4% agreed or strongly agreed that they did not know what might happen next, while only 24.8% were of the belief they would be harmed.

**Figure 3:**
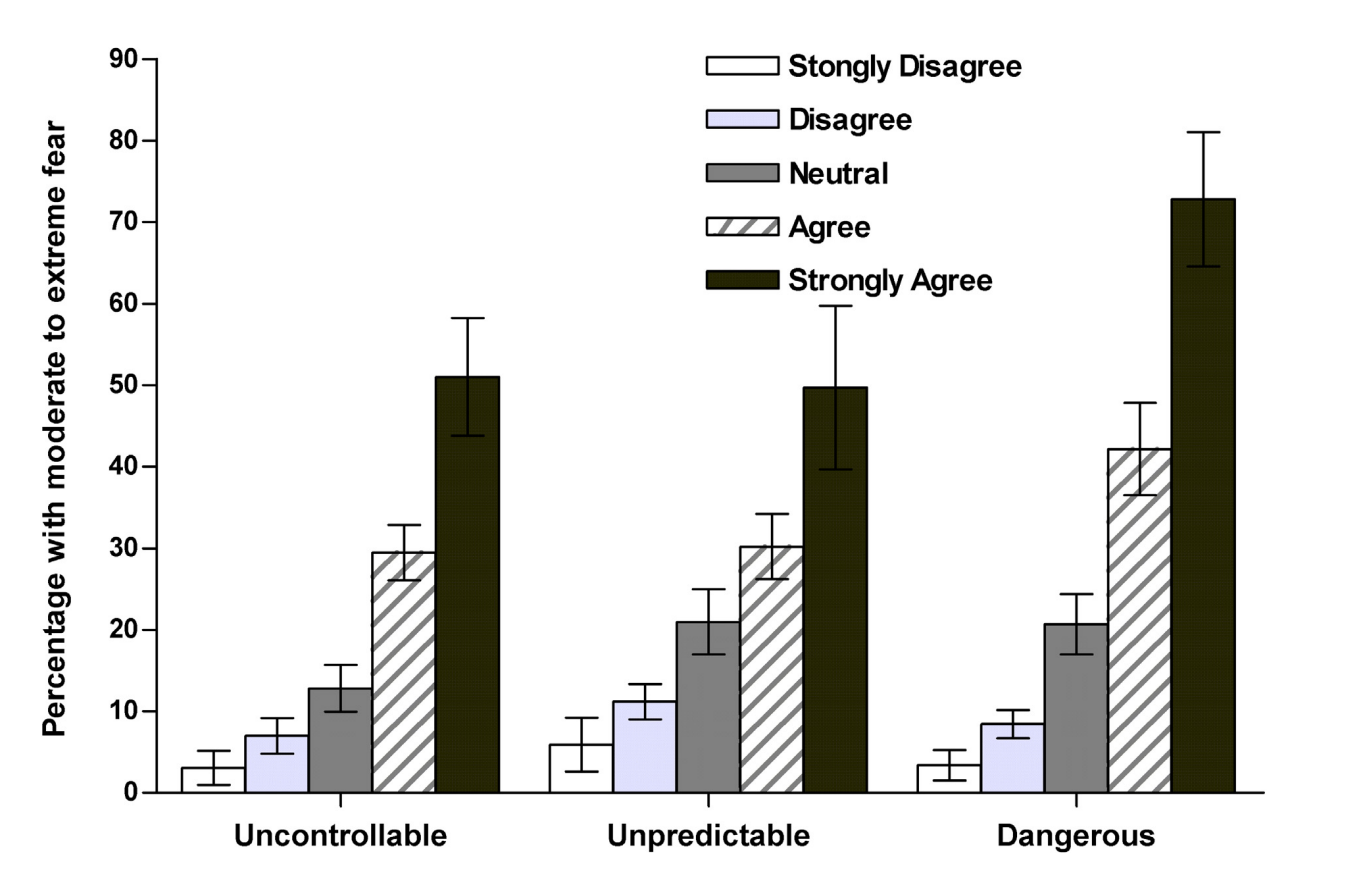
Prevalence (and 95% CI) of participants with high dental fear (moderate to extreme) by perceptions of uncontrollability, unpredictability and dangerousness.

The relationships between dental fear and perceptions of uncontrollability, unpredictability and dangerousness are shown in Figure 3. Only 3–6% of people who perceived the dental visit as being highly controllable, highly predictable and highly safe had moderate to extreme dental fear. However, the prevalence of high dental fear among people who regarded the dental environment as being highly uncontrollable, highly unpredictable or highly dangerous was 51.0%, 49.8% and 72.9% respectively. All three variables showed a strong linear association with dental fear. Univariate analysis of variance confirmed that dental fear was significantly associated with perceptions of uncontrollability (*F *= 81.22, *p *< 0.001), perceptions of unpredictability (*F *= 40.93, *p *< 0.001) and with perceptions of the likelihood of harm (*F *= 107.11, *p *< 0.001).

In an effort to identify possible confounders for subsequent multivariate analyses, analysis of variance was used to examine the association between dental fear and a number of demographic and socio-economic variables which have previously been found to have a relationship with dental fear [13]. Age was significantly associated with dental fear (*F *= 13.29, *p *< 0.001), with the prevalence of high dental fear increasing from approximately 10% of 15–24-year-olds to 27.1% of middle aged adults (age 40–64) before reducing to 7.6% of the oldest age group (Table 2). Sex was also significantly associated with dental fear, with more females (prevalence = 26.1%) reporting high dental fear than males (prevalence = 14.3%), *F *= 41.30, *p *< 0.001. No significant difference in the prevalence of high dental fear was noted across either income categories or employment types.

**Table 2:** Prevalence of high dental fear (SD and 95% confidence intervals) by demographic and socio-economic characteristics

Characteristic	*n*	Prevalence	SD	95% CI
Age*				
15–24	593	9.97	29.98	4.74,15.19
25–39	1,104	17.27	37.81	13.77,20.76
40–64	1,699	27.10	44.46	24.61,29.58
65–79	455	19.35	39.55	15.46,23.25
80+	81	7.64	26.73	0.00,15.29
Sex*				
Male	1,880	14.33	35.05	11.79,16.88
Female	2,052	26.07	43.91	23.74,28.40
Income				
<$20,000	488	24.44	43.02	20.31,28.57
$20,000–$39,999	766	20.69	40.54	17.50,23.88
$40,000–$59,000	676	23.04	42.14	18.58,27.50
$60,000–$79,000	564	19.35	39.54	14.44,24.25
$80,000+	1,041	19.36	39.53	15.46,23.26
Employment status				
Unemployed	583	20.60	40.48	15.98,25.21
Part-time	866	22.54	41.81	18.84,26.24
Full-time	1,549	20.70	40.53	17.40,24.00
Student/retired	756	20.61	40.48	17.33,23.89

* *p *< 0.001

To test for the significance of the associations between dental fear and the three vulnerability-related variables after controlling for the possible confounding variables, a logistic regression analysis was undertaken with dental fear ('none or little'/'moderate to extreme') as the dependent variable. Age and sex were entered into the model as these were significantly associated with dental fear in the bivariate analyses. Overall, the model accounted for just over one-third of the variance in high dental fear, with a Nagelkerke *R*^2 ^of 0.36. The summary output from the regression analysis is shown in Table 3. After controlling for all variables in the analysis, only perceived uncontrollability and perceived dangerousness showed significant independent associations with dental fear. People who strongly agreed that they felt out of control when in the dental chair had 6.81 the odds of being moderately to extremely afraid, in comparison to people who strongly disagreed that they felt out of control when in the dental chair. The association with perceived dangerousness was even stronger, with those people strongly believing they would be harmed having 29.13 the odds of having high dental fear compared to people who strongly believed that they would not be harmed while in the dental chair.

**Table 3:** Parameter coefficients, adjusted odds ratios (ORs) and 95% confidence intervals of cognitive vulnerability variables, demographic and socio-economic characteristics on moderate to extreme dental fear

Variable	B	Sig.	OR	95% CI
Uncontrollability				
Strongly Disagree			Ref.	
Disagree	0.35	0.145	1.42	0.89,2.29
Neutral	1.01	<0.001	2.76	1.60,4.75
Agree	1.27	<0.001	3.56	2.01,6.30
Strongly Agree	1.92	0.001	6.81	2.29,20.26
Unpredictability				
Strongly Disagree			Ref.	
Disagree	-0.11	0.714	0.90	0.51,1.58
Neutral	-0.23	0.490	0.80	0.42,1.52
Agree	0.08	0.794	1.08	0.60,1.96
Strongly Agree	-0.14	0.789	0.87	0.31,2.43
Dangerousness				
Strongly Disagree			Ref.	
Disagree	1.03	<0.001	2.79	1.66,4.69
Neutral	1.86	<0.001	6.45	3.93,10.59
Agree	2.73	<0.001	15.38	9.06,26.11
Strongly Agree	3.37	<0.001	29.13	12.45,68.15
Age				
80+			Ref.	
65–79	0.67	0.331	1.95	0.51,7.54
40–64	1.02	0.132	2.76	0.74,10.35
25–39	0.44	0.523	1.55	0.41,5.89
15–24	-0.48	0.520	0.62	0.15,2.66
Sex				
Male			Ref.	
Female	0.83	<0.001	2.28	1.74,3.00

Following from the results of the logistic regression it was suspected that high correlations between the three cognitive vulnerability variables may explain why perceptions of unpredictability were not found to have a significant independent association with dental fear. Indeed, Spearman's rho correlation coefficients were 0.64 between uncontrollability and unpredictability, 0.50 between uncontrollability and dangerousness, and 0.50 between unpredictability and dangerousness, with all correlations being significant at *p *< 0.001. In an attempt to tease apart the associations, another series of logistic regression models were run. First, unpredictability was entered with just age and sex. People who perceived being in a dental chair as being highly unpredictable were much more likely to have high dental fear than people who perceived the dental visit as being highly predictable (OR = 23.2, *p *< 0.001). All possible responses were significant when compared to the reference category 'Strongly disagree'. Two more models were then tested, the first with unpredictability entered with age, sex and uncontrollability and the second with unpredictability entered with age, sex and dangerousness. In both cases, while unpredictability was significantly associated with dental fear, the corresponding odds ratios were considerably reduced. In addition, only those responding 'Agree' or 'Strongly Agree' had significant odds of high dental fear compared to the reference category 'Strongly disagree'. It appears likely then, that unpredictability did not have a significant independent association with dental fear (Table 3) because of its high relatedness to both dangerousness and uncontrollability.

To test whether the associations obtained in the multivariate analyses were consistent using a different dichotomisation of the dependent variable, results were rerun using dental fear categorised as Not at all/A little/Moderately and Very/Extremely. While the lower prevalence of 'high fear' meant that confidence intervals were correspondingly larger and there was greater overlap of 95% confidence intervals, the exact pattern of results were obtained under this condition, and the statistical significance of the main effects remained unaltered.

## Discussion

This study found that perceptions of uncontrollability, unpredictability and dangerousness had appreciable bivariate associations with dental fear. However, only perceived uncontrollability and dangerousness had a significant association with dental fear after controlling for the other vulnerability variables and age and sex. The substantial bivariate associations between dental fear and perceptions of uncontrollability, unpredictability and dangerousness are consistent with the Cognitive Vulnerability Model of the etiology of fear which proposes that how an individual perceives a situation is a crucial determinant of fear in that situation. While few people who perceived being in a dental chair as being highly controllable, highly predictable and highly safe had moderate to extreme dental fear, approximately 50% or more of people who regarded the dental environment as being either highly uncontrollable, highly unpredictable or highly dangerous had high dental fear.

Many studies have looked at the variables of control, danger and, to a lesser extent, predictability in relation to dental fear so it is perhaps not surprising that these perceptual characteristics of the dental environment showed an association with dental fear. Milgrom, for instance, identified lack of control as a primary concern of dental phobics [33] and a number of studies have confirmed its importance. For example, Milgrom and colleagues found that the probability of a stressful experience resulting in fear and avoidance is enhanced when perceptions of control are low [34]. In addition, studies by Logan and colleagues have demonstrated the association between dental fear and a combination of both a high desire for control and low perceived control in dental fear [35,36]. Consistent with these findings, dental patients with high desire for control, but who perceive little actual control, report higher levels of expected pain before treatment than do other patient subgroups [37]. The importance of control as an issue in dental fear has also been acknowledged in the development of belief scales. The Dental Beliefs Survey [33], for example, which has 'lack of control' as one of the identified factors [38], has been reported as being highly related to dental fear [39]. More specifically, the Revised Iowa Dental Control Index, which measures both desired and predicted control in dental situations, has been found to correlate significantly with dental fear [40]. In relation to depression, it has been speculated that perceptions of uncontrollability related to an aversive event may be more important than the event per se [41], and the same may be the case for anxiety disorders such as dental phobia.

Concerns over pain and harm when attending a dentist often revolve around the receipt of injections and use of the drill [42,43]. However, even non-invasive procedures such as prophylaxis may be considered painful or unpleasant [44]. Although there has been an increasing emphasis placed on the importance of non-invasive dentistry, many patients are still concerned by the level of pain and discomfort associated with visiting the dentist and this is underlined by the strong association between dental fear and concerns about being harmed found in this study. While approaches such as the iatrosedative technique, which relies on the dentist's promises and actions to protect the patient from what is perceived as dangerous [45,46], can address patient concerns of expected pain, there is no evidence that the use of these techniques is prevalent in dentistry at this time.

While many studies have previously looked at the association between dental fear and control, the major focus of most of this research has been on the use of control as an intervention to modify fear once it is already established rather than on control, or lack of, as an antecedent of fear. Whereas many researchers and clinicians accept that affording patients some control over the dental environment reduces fear and anxiety, the Cognitive Vulnerability Model places perceived lack of control as a core cognitive variable in the etiology of fear. Similarly, it is the perception of dangerousness associated with going to a dentist which is relevant to the cause of dental fear rather than whether or not a person has experienced a painful or traumatic incident per se.

Little research has looked at the role of unpredictability in relation to dental fear although it does make intuitive sense that such an association would exist. Dental patients can not view what is happening in their own mouth and are for the most part unable to anticipate when pain might occur. If the dentist does not adequately explain the procedure, the patient may also find the treatment process to be unpredictable, adding to his or her fear. Indeed, it is quite likely that lack of predictability and controllability, coupled with anxiety over pain, operate in combination, each adding to the effect of the other to generate the fear response. However, this study found no independent association between perceptions of predictability and dental fear in the multivariate analyses in this paper, despite a strong bivariate association. Presumably the reason for this was because perceptions of uncontrollability, dangerousness and unpredictability all overlapped to a considerable extent. Despite the failure of this study to find a statistical association between perceptions of unpredictability and dental fear, it is still possible from an individual person's point of view that all the vulnerability-related perceptions would need to be addressed to effectively alleviate dental fear.

While this study looked at perceptions of uncontrollability, unpredictability and dangerousness, the fourth characteristic comprising the vulnerability-related dental schema, disgust, was not examined. In general, there has been very little research investigating the role of disgust in the fear of dental visits, although there is some evidence that it may relate to issues with the smell associated with dental surgeries [47] and fear of contamination with germs or disease [48]. Certainly there is a strong association between disgust and fears and phobias relating to blood, injections and injuries [49] and this may be relevant to the experience of some dental procedures. In reality, however, it may be the case that disgustingness has less of a role in the etiology of dental fear than perceptions of uncontrollability, unpredictability and dangerousness. The Cognitive Vulnerability Model proposes that fear of any given stimulus is related to a specific combination of the vulnerability variables. Whereas some fears may centre more on disgust (e.g. fear of maggots or slugs) others may centre more on the other variables, or indeed each perceptual characteristic may contribute relatively equally. Nonetheless, further investigation of the role of disgust in dental fear is warranted as this may be a salient characteristic of the dental fear experience for some people.

An important aspect of this study was that all participants had undergone a dental examination prior to receiving the self-complete questionnaire. Because people with high dental fear or who have dental phobias are less likely to attend a dental examination, it is quite probable that a number of high fear individuals may have dropped out from the study at the point of the examination. This would have resulted in fewer people with high dental fear and may have impacted on the statistical analyses. It is likely that stronger effects would have been found had those extra high-fear individuals been questioned. The fact that all participants had recently attended the associated dental examination also means that a person's dental fear and their perceptions of uncontrollability, unpredictability and dangerousness associated with dental visits may have been influenced by the examination process. In particular, no treatment was provided and great care was taken by examiners to explain all procedures prior to carrying them out, which may have effected both perceptions of dangerousness and perceptions of unpredictability respectively.

While the actual prevalence of dental fear in the population is unknown, there is good quality census data on numerous other population characteristics. Despite weighting of the data by age and sex, comparisons of the study sample with national census data indicated that the study sample had higher percentages of people who were unemployed or were employed part time, and a higher percentage of people with higher incomes. While moderate participation rates in health studies do not necessarily mean that there are errors in producing population estimates [50], it is almost inevitable that small biases in terms of population characteristics may be observed. However, while there may be some differences in socioeconomic characteristics between the study sample and the population, it should be noted that a separate analysis of the national survey data used for this study indicated that the extent of participation bias in terms of oral health indicators was small [51] and that this study actually found no significant effect for either employment status or income on dental fear.

One of the limitations of this study is the use of a single-item measure of dental fear and of single-item measures of uncontrollability, unpredictability and dangerousness. While single-item measures of dental fear often correlate with multi-item scales and may show good sensitivity and specificity [52], kappa statistics indicating agreement corrected for chance show only fair to moderate agreement [53]. General fear items are also incapable of picking up the many nuances of what is a multifactorial condition. Similarly, further work in this area requires the development of a more extensive set of questions assessing perceptions of uncontrollability, unpredictability, dangerousness and disgustingness. Similar scales which have been developed for spiders and other animals [27] should ideally be employed for investigations of dental fear.

Another limitation of the study is the use of a cross-sectional design which does not allow for a determination of cause and effect. However, given that this paper is intended as a preliminary investigation of the Cognitive Vulnerability Model as it applies to dental fear, the cross-sectional data provide a useful initial confirmation of the existence of relationships between dental fear and vulnerability-related perceptions. Indeed, and despite the methodological limitations of cross-sectional studies, there are important clinical implications stemming from the results of this study. The model of fear underlying this investigation holds that vulnerability-related perceptions forming a cognitively active schema comprise the core constructs of dental fear. Clinicians need to be aware not only of their patients' level of anxiety and fear but also of their perceptions of uncontrollability, unpredictability, dangerousness and disgustingness in the dental environment. While there has been no work yet conducted with dental fear, experimental studies of other specific fears indicate that altering a person's perception of control, predictability and safety can significantly affect their level of fear [29,30]. It would be important in relation to dental fear to test these associations, and determine the direction of causality, using a more appropriate longitudinal or experimental design in the future.

## Conclusion

This paper presents findings linking perceptions of uncontrollability, unpredictability and dangerousness to fear of going to the dentist and provides preliminary support for the use of the Cognitive Vulnerability Model in better understanding the phenomenology of dental fear. An understanding of vulnerability-related perceptions is important as these perceptions relate to key areas where effort should be directed to prevent or alleviate an individual's dental fear. It has long been acknowledged that affording dental patients a sense of control and freedom from pain and discomfort can limit or help manage dental fear. However, the Cognitive Vulnerability Model provides a theoretical framework on which to base and further explore these suggestions and recommendations. Given the high prevalence of dental fear in the community and the detrimental social, psychological and physical consequences accompanying the avoidance of dental care, there is a pressing need to unravel the issues at the core of this problem.

## Competing interests

The author(s) declare that they have no competing interests.

## Authors' contributions

JA conceived of the paper, performed the statistics analyses and wrote the manuscript. GDS and AJS designed and implemented the National Survey of Adult Oral Health and helped write the manuscript. All authors have read and approved the final manuscript.

## Pre-publication history

The pre-publication history for this paper can be accessed here:


